# Enhanced Detection of Landmark Minimal Residual Disease in Lung Cancer Using Cell-free DNA Fragmentomics

**DOI:** 10.1158/2767-9764.CRC-22-0363

**Published:** 2023-05-30

**Authors:** Siwei Wang, Zhijun Xia, Jing You, Xiaolan Gu, Fanchen Meng, Peng Chen, Wanxiangfu Tang, Hua Bao, Jingyuan Zhang, Xue Wu, Yang Shao, Jie Wang, Xianglin Zuo, Lin Xu, Rong Yin

**Affiliations:** 1 Department of Thoracic Surgery, Jiangsu Key Laboratory of Molecular and Translational Cancer Research, Nanjing Medical University Affiliated Cancer Hospital & Jiangsu Cancer Hospital & Jiangsu Institute of Cancer Research, Nanjing, P.R. China.; 2 Department of Anesthesiology, Nanjing Medical University Affiliated Cancer Hospital & Jiangsu Cancer Hospital & Jiangsu Institute of Cancer Research, Nanjing, P.R. China.; 3 Nanjing Geneseeq Technology Inc., Nanjing, Jiangsu, P.R. China.; 4 Department of Science and Technology, Nanjing Medical University Affiliated Cancer Hospital & Jiangsu Cancer Hospital & Jiangsu Institute of Cancer Research, Nanjing, P.R. China.; 5 Biobank of Lung Cancer, Jiangsu Biobank of Clinical Resources, Nanjing, P.R. China.; 6 Collaborative Innovation Center for Cancer Personalized Medicine, Nanjing Medical University, Nanjing, P.R. China.

## Abstract

Currently, approximately 30%–55% of the patients with non–small cell lung cancer (NSCLC) develop recurrence due to minimal residual disease (MRD) after receiving surgical resection of the tumor. This study aims to develop an ultrasensitive and affordable fragmentomic assay for MRD detection in patients with NSCLC. A total of 87 patients with NSCLC, who received curative surgical resections (23 patients relapsed during follow-up), enrolled in this study. A total of 163 plasma samples, collected at 7 days and 6 months postsurgical, were used for both whole-genome sequencing (WGS) and targeted sequencing. WGS-based cell-free DNA (cfDNA) fragment profile was used to fit regularized Cox regression models, and leave-one-out cross-validation was further used to evaluate models’ performance. The models showed excellent performances in detecting patients with a high risk of recurrence. At 7 days postsurgical, the high-risk patients detected by our model showed an increased risk of 4.6 times, while the risk increased to 8.3 times at 6 months postsurgical. These fragmentomics determined higher risk compared with the targeted sequencing–based circulating mutations both at 7 days and 6 months postsurgical. The overall sensitivity for detecting patients with recurrence reached 78.3% while using both fragmentomics and mutation results from 7 days and 6 months postsurgical, which increased from the 43.5% sensitivity by using only the circulating mutations. The fragmentomics showed great sensitivity in predicting patient recurrence compared with the traditional circulating mutation, especially after the surgery for early-stage NSCLC, therefore exhibiting great potential to guide adjuvant therapeutics.

**Significance:**

The circulating tumor DNA mutation-based approach shows limited performance in MRD detection, especially for landmark MRD detection at an early-stage cancer after surgery. Here, we describe a cfDNA fragmentomics–based method in MRD detection of resectable NSCLC using WGS, and the cfDNA fragmentomics showed a great sensitivity in predicting prognosis.

## Introduction

Non–small cell lung cancer (NSCLC) is the leading cause of worldwide cancer-related deaths (1). After surgical resection of the tumor, approximately 30%–55% of the patients with NSCLC eventually develop recurrence due to minimal residual disease (MRD; refs. 1, 2). Up-to-date, Guardant 360 and FoundationOne Liquid CDx, which focused on mutation detection in 73 and 311 genes, remain the only two liquid cell-free DNA (cfDNA) assays approved by the FDA for utilization in NSCLC (3, 4). However, they both suffer from low sensitivity and are unsuitable for detecting MRD. Therefore, there is an urgent clinical need to predict the recurrence risk for patients with postsurgical NSCLC accurately.

Tumor cells are known to shed DNAs into circulation, which shares the same mutational profile, and therefore can be used to develop noninvasive liquid biopsy assays (2, 5–11). The low abundance of circulating tumor DNA (ctDNA) in patients’ plasma samples remains the biggest obstacle to detecting MRD through ctDNA mutation profiling. This is even more challenging for postsurgery landmark samples, which are collected days after the tumor resection, as they often yield extremely low ctDNA content (<0.1%; refs. 5, 6). Abbosh and colleagues designed a patient-specific targeted sequencing panel for ctDNA mutation–based MRD detection (6). However, their panel suffered from low sensitivity, especially at landmark postsurgery, yielding only 36% sensitivity at 90% specificity (6). Chen and colleagues demonstrated a limited performance in detecting landmark MRD (44% sensitivity at 88% specificity) using their circulating single-molecule amplification and resequencing technology assay (9), which was more similar to the results by Abbosh and colleagues (6). Moreover, Zhang and colleagues investigated the utilization of MRD detection assay in a comprehensive cohort of 261 patients with NSCLC, yet only showing a 36.2% sensitivity at 98.0% specificity for MRD detection at landmark (7). Likewise, Waldeck and colleagues 2022 reported a 40% MRD detection rate using 1–2 weeks of postsurgical samples from a relatively small cohort of 20 patients with resectable NSCLC (12). Li and colleagues 2021 reported only a 23% positive MRD detection rate while using the somatic mutation approach on the landmark plasma samples (13). A similar sensitivity (21.4%) was observed by Xia and colleagues in the LUNGCA-1 study, which detected MRD in 15 of 70 patients with progression using 3 days of postsurgical samples (14). Gale and colleagues also showed a 45.4% sensitivity for landmark MRD detection in a cohort of 88 patients with NSCLC using a whole-exome sequencing approach (8).

Overall, the ctDNA mutation–based approach shows limited sensitivity in MRD detection, especially for landmark MRD detection at an early-stage cancer after surgery. Such limitation is potentially contributed by the combination of low ctDNA abundance and relatively low sensitivity of the target sequencing approach (15–17). The whole-genome sequencing (WGS) approach, however, has shown a higher sensitivity as it is not limited by the number of ctDNA molecules and their specific location (16). Mathios and colleagues showed that fragmentomics machine learning modeling using WGS data could detect patients with NSCLC with extremely high sensitivity (95%). Moreover, Bao and colleagues developed an ultrasensitive assay for multi-cancer early detection incorporating plasma cfDNA fragmentomic features derived from WGS data, showing a 90.5% sensitivity at 95.5% specificity for detecting very early-stage lung cancer (17). A recent article by Wang and colleagues reported an integrated model utilizing five machine learning algorithms on five different cfDNA fragmentomics features for detecting early-stage lung cancer. The integrated model yielded high AUCs in both validation cohorts (0.984 and 0.987; ref. 18). Furthermore, Zviran and colleagues showed an excellent 100% sensitivity while detecting MRD in a small cohort of 22 patients with WGS, albeit limited by a relatively low specificity of 71% (19). Therefore, fragmentomics profiling may have great potential in MRD detection.

In this study, we aim to explore the utilization of plasma cfDNA fragmentomic profiling for MRD detection, especially for landmark detection. We developed a cohort of 100 patients with NSCLC who received curative tumor resections as the standard of care. Fragmentomics feature profiling was retrieved on the basis of WGS data using plasma samples collected at both landmark and longitudinal timepoints, defined as around 7 days and 6 months postsurgery, respectively. Machine learning models were evaluated and then constructed using these fragmentomics profiles.

## Materials and Methods

### Patient Enrollment and Sample Collection

A total of 100 patients with NSCLC were initially enrolled in this study cohort between April 2017 and January 2019. All study protocols were approved by the ethics committee of Jiangsu Cancer Hospital and in accordance with the Declaration of Helsinki. Written informed consents were provided by all patients.

**FIGURE 1:**
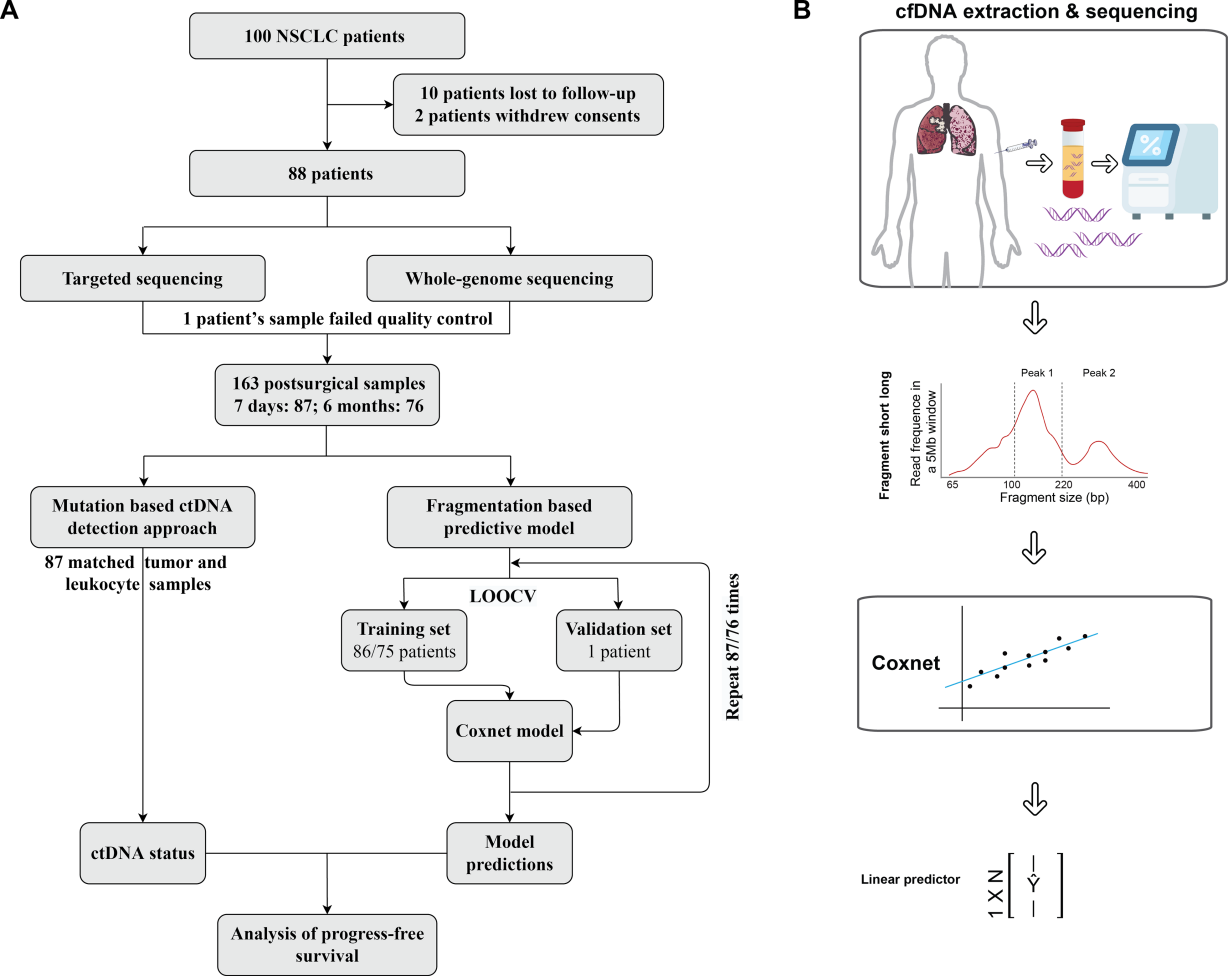
Study design and flow chart of methodology. **A,** Cohort and study design. **B,** Fragmentomics feature extraction and modeling details.

Among the 100 enrolled patients, 2 patients later withdrew consents and 10 patients were lost during the follow-up period, resulting in a cohort size of 88 patients (Fig. 1A). These patients were pathologically diagnosed with NSCLC (AJCC, 8th edition) and received curative tumor resections as the standard of care. The histologic subtype and tumor–node–metastasis staging were identified and reviewed by two pathologists. Postsurgical plasma samples, which were collected were scheduled at 7 days and 6 months after surgeries, were used for both target sequencing (425 cancer-associated gene panel, GeneseeqPrime) and WGS. Primary tumor tissue samples, as well as paired leukocyte samples, were also collected and sequenced by the 425-gene panel. One patient was removed from the cohort due to the failure of plasma samples during the quality control process.

### Library Preparation and Sequencing

Genomic DNA from tissue samples was extracted with QIAamp DNA formalin-fixed paraffin-embedded (FFPE) Tissue Kit, while DNeasy Blood & Tissue Kit (Qiagen) was used for plasma samples. The Qubit 3.0 fluorometer and dsDNA HS Assay Kit (Thermo Fisher Scientific) were used to quantify the extracted DNA, following the manufacturer's instruction. A deparaffin procedure using xylene was performed on FFPE samples before the genomic DNA extraction with QIAamp DNA FFPE Tissue Kit (Qiagen) per manufacturer's instruction. To extract genomic DNA from the plasma samples, centrifugation was performed at high speed to remove any cell debris. The supernatant was then used for the cfDNA extraction using QIAamp Circulating Nucleic Acid Kit (Qiagen) per manufacturer's instruction. Library preparations were performed with KAPA Hyper Prep Kit (KAPA Biosystems) according to manufacturer's suggestions for different sample types.

For target sequencing, sequential operations of end-repairing, A-tailing, and indexed adapter ligation were performed to 6.08–200 ng (median: 70.5 ng) of cfDNA or 1 μg of fragmented genomic DNA, followed by size selection using Agencourt AMPure XP beads (Beckman Coulter). Hybridization-based target enrichment was carried out with GeneseeqPrime pan-cancer gene panel (425 cancer-relevant genes), which covers a total of approximately 1.5 Mb genomic regions (including ∼0.93 Mb coding regions) using approximately 20,000 probes, and xGen Lockdown Hybridization and Wash Reagents Kit (Integrated DNA Technologies). Captured libraries were on-beads PCR amplified with Illumina p5 (50 AAT GAT ACG GCG ACC ACC GA 30) and p7 primers (50 CAA GCA GAA GAC GGC ATA CGA GAT 30) in KAPA HiFi HotStart ReadyMix (KAPA Biosystems), followed by quantification by qPCR using the KAPA Library Quantification Kit (KAPA Biosystems) and purification using Agencourt AMPure XP beads. Library fragment size was assessed by Bioanalyzer 2100 (Agilent Technologies). Sequencing of the target-enriched library was then performed on Illumina HiSeq4000 platform using PE150 sequencing chemistry (Illumina).

WGS libraries were constructed using the KAPA Hyper Prep Kit (KAPA Biosystems) according to the manufacturer's protocol. In brief, 5–10 ng of cfDNA per sample was subjected to end-repairing, A-tailing, and ligation with adapters sequentially. The Hamilton Microlab STAR automated liquid handling platform (Hamilton Company) was used for the automated pipeline. The libraries were quantified with the KAPA SYBR FAST qPCR Master Mix (KAPA Biosystems) and underwent paired-end sequencing on NovaSeq platforms (Illumina) according to the manufacturer's instructions. PE150 was used as it was shown by Cristiano and colleagues 2019 that the peak frequency of cfDNA fragment size was around 167 bp (20).

### Fragmentomics Machine Learning Model

The raw reads for the WGS data were first trimmed (trailing reads only, quality <20) with Trimmomatic (21). The Picard toolkit (http://broadinstitute.github.io/picard/) was then used for PCR duplicates removal. The reads were then mapped onto the human reference genome (GRCh37/UCSC hg19) using the sequence aligner Burrows-Wheeler Aligner (BWA, v0.7.12; ref. 22). Fragment size was calculated using the mapped distances between 5′ ends of the read pairs and should not be significantly impacted by the trimming of trailing reads. To eliminate the potential impact on the predictive power of the different coverages among the WGS data, we downsampled the coverages to a unified 5 ×.

The Fragment Size Ratio (FSR) profile, which was adapted from previous reports by Ma and colleagues and Zhang and colleagues (23, 24), examined the ratios of different size fragments across the human genome, as the cfDNA fragments are reported to be aberrantly short and long in cancer patient's plasma samples (25). In-house scripts were used to construct these fragmentation profiles as reported previously (23, 24), which focused on comparing the short/long reads around the first and second peaks, as shown in Fig. 1B. The ShortPeak1, LongPeak1, ShortPeak2, and LongPeak2 fragments were defined as 100–150 bp, 151–220 bp, 221–300 bp, and 311–400 bp, according to the overall fragment length profile in our cohorts (Fig. 1B). The ratios of the short/long fragments of both peaks for each sample were examined in 5 Mb bins, resulting in a total of 2,164 (541 bins × 4) FSR features from the 541 bins genome-wide, excluding the sex chromosomes (22 autosomes) and masking any repeat regions, which were then used by the machine learning algorithms for model construction. Following the previous report by Cristiano and colleagues, a local smoother was applied to remove any potential bias in the coverage due to the guanine-cytosine (GC) content (20).

A total of 87 patients with NSCLC, including 23 patients with recurrence during follow-up, were used to construct predictive models for MRD. Two machine learning models, which used either 7 days or 6 months postsurgical samples, were constructed on the basis of the Penalized Cox Models (Coxnet) algorithm by the scikit-survival package (0.17.2; ref. 26). The Coxnet algorithm was selected for its advantages against other competing methods, including efficacy, stability, significantly shorter runtime and, most importantly, its ability to process high-dimensional input features (27). Both models used leave-one-out cross-validation to evaluate the predictive performance of MRD. As shown in Fig. 1A, each of the samples (87 and 76 for the 7 days and 6 months postsurgical samples, respectively) were used once as a validation set during the leave-one-out cross-validation. The remaining samples (86 or 75) were used as the training set to fit a Coxnet model, which were then used for predicting the MRD risk score for the validation set. This process was then repeated 87 (76) times till the risk score for every sample was generated. To determine the cutoffs for fragmentomics-predicted MRD-positive status, ROC curves were constructed by the pROC package (v. 1.17.0.1) using the sample risk scores. The cutoffs for both models were defined as the best sensitivity at a minimal of 90% specificity.

### Identification of ctDNA Mutation Profile

The ctDNA mutation profile was constructed using a previously reported method (2, 10) by tracking somatic mutations identified from cancer tissue samples in patients’ plasma samples. Variant calling of plasma samples was tumor-informed by comparing the identified mutations against mutations detected in the primary tumor tissue sample and the leukocyte samples. Tissue clonality was annotated for each variant detected in plasma. Somatic single-nucleotide variant (sSNV) and insertion/deletion (InDel) detected in plasma samples were filtered if (i) they were not in the paired tissue mutational profile, (ii) they fell in an in-house list of clonal hematopoiesis variants, or (iii) they were detected in paired leukocyte controls with at least one variant read. Furthermore, a normal pool was constructed using 100 healthy individuals to determine whether the mutation found in plasma samples was significantly higher than background noise.

Trimmomatic was used for FASTQ file quality control. Leading/trailing low quality (quality reading below 20) or N bases were removed. Paired-end reads were then aligned to the reference human genome (build hg19) using the BWA (v0.7.12; ref. 22). Local realignment around indels and base quality score recalibration was performed with the Genome Analysis Toolkit (GATK 3.4.0; ref. 28). Cross-sample contamination was estimated using ContEst (Broad Institute; ref. 29). Briefly, ContEst quantifies contamination in next-generation sequencing data by identifying homozygous nonreference SNPs in the 1000 Genomes database and assessing the likelihood of observing alternate alleles at these genomic locations in the sequencing data. sSNV and InDel calling was performed using Vardict (30).

### Statistical Analysis

The sensitivity/recall [True Positive/(TP + False Negative)], specificity [True Negative/(TN + False Positive)], positive predict value/precision [TP/(TP+FP)], and negative predict value [TN/(TN+FN)] as well as the 95% confidence interval (CI), were calculated using the epiR package (v 2.0.19). All statistical analyses were performed in R (v.4.1.3).

### Availability of Data and Materials

The sequencing data reported in the study have been deposited in the European Genome-phenome Archive (EGA) database as EGAD00001010300. The data are deposited under controlled access for access to the data contact Dr. Rong Yin, rong_yin@njmu.edu.cn. All the other data supporting the findings of this study are available within Supplementary Data and from the corresponding author upon reasonable request.

## Results

### Participant Characteristics in the Cohort

A total of 100 patients with pathologically diagnosed NSCLC, all received curative tumor resections as the standard of care. Among these 100 patients, 10 patients were lost during the follow-up period, while 2 patients withdrew their consents and 1 patient's samples failed the quality-control process, resulting in a final cohort size of 87 (Fig. 1). All patients had not received neoadjuvant therapy.

As shown in Supplementary Table S1, the median age for this cohort is 62.1 years (42–79 years). The final cohort consisted of 46 males (52.9%) and 41 females (47.1%). In terms of histology, adenocarcinomas contributed the majority of cases (72/87, 82.8%), followed by squamous cell carcinomas (11/87, 12.6%), while 4.6% of the patients had other types of patho-histologies. As shown in Table 1, a multivariate Cox regression confirmed that the different histology types have no significant impact on our purpose of developing an ultrasensitive and affordable fragmentomic assay for MRD detection in patients with NSCLC (HR = 0.958, 95% CI: 0.293–3.133, *P* = 0.9434). The cohort contained 42 stage I, 14 stage II, and 31 stage III patients, while 43.7% (38/87) developed lymph node metastasis. It was also noted that the majority (81.6%, 71/87) of these patients were nonsmokers. For the 23 patients who developed recurrence, the median follow-up time was 311 days (106–943 days), while the median follow-up time for the 64 recurrence-free patients was 875 days (661–1,259 days).

**TABLE 1:** Univariate and multivariate Cox analysis of risk factors

7 days postsurgery	No. of patients	HR (95% CI)		6 months postsurgery	No. of patient	HR (95% CI)	
		Univariate	Multivariate			Univariate	Multivariate
**Fragmentation**				**Fragmentation**			
**Low risk**	71	Reference	Reference	**Low risk**	60	Reference	Reference
**High risk**	16	4.587 (1.998–10.53)	3.300 (1.228–8.866)	**High risk**	16	8.285 (3.298–20.81)	6.967 (2.217–21.890)
**Mutation**				**Mutation**			
**Negative**	77	Reference	Reference	**Negative**	67	Reference	Reference
**Positive**	10	3.077 (1.212–7.81)	2.742 (0.650–11.567)	**Positive**	9	6.209 (2.301–16.76)	2.096 (0.602–7.297)
**Age**				**Age**			
	87	1.027 (0.976–1.081)	1.013 (0.946–1.084)		76	1.024 (0.969–1.082)	1.034 (0.956–1.119)
**Gender**				**Gender**			
**Female**	41	Reference	Reference	**Female**	39	Reference	Reference
**Male**	46	2.976 (1.172–7.554)	2.920 (0.777–10.976)	**Male**	37	2.649 (1.006–6.976)	1.488 (0.436–5.080)
**Smoking history**				**Smoking history**			
**No**	71	Reference	Reference	**No**	62	Reference	Reference
**Yes**	16	2.953 (1.25–6.977)	1.808 (0.563–5.801)	**Yes**	14	2.295 (0.87–6.054)	0.834 (0.240–2.898)
**Adjuvant therapy**				**Adjuvant therapy**			
**No**	52	Reference	Reference	**No**	41	Reference	Reference
**Yes**	34	1.716 (0.7563–3.893)	0.895 (0.342–2.343)	**Yes**	34	2.246 (0.8826–5.715)	0.901 (0.258–3.149)
**Stage**				**Stage**			
**I**	42	Reference	Reference	**I**	35	Reference	Reference
**II**	14	4.398 (1.179–16.41)	3.537 (0.792–15.787)	**II**	13	4.404 (0.985–19.7)	3.382 (0.548–20.871)
**III**	31	5.499 (1.806–16.74)	3.52 (0.893–14.132)	**III**	28	5.735 (1.614–20.38)	3.895 (0.710–21.367)
**Histology**				**Histology**			
**Adenocarcinoma**	72	Reference	Reference	**Adenocarcinoma**	62	Reference	Reference
**Squamous cell carcinoma**	11	3.693 (1.418–9.616)	0.958 (0.293–3.133)	**Squamous cell carcinoma**	11	4.107 (1.527–11.050)	1.946 (0.558–6.804)

### Fragmentomics Coxnet Models for MRD Detection

We developed two Coxnet models by using the FSR feature profile of both 7 days and 6 months postsurgical to predict the risk status for recurrence. ROC curves were constructed with predicted risk scores from leave-one-out cross-validation results.

**FIGURE 2:**
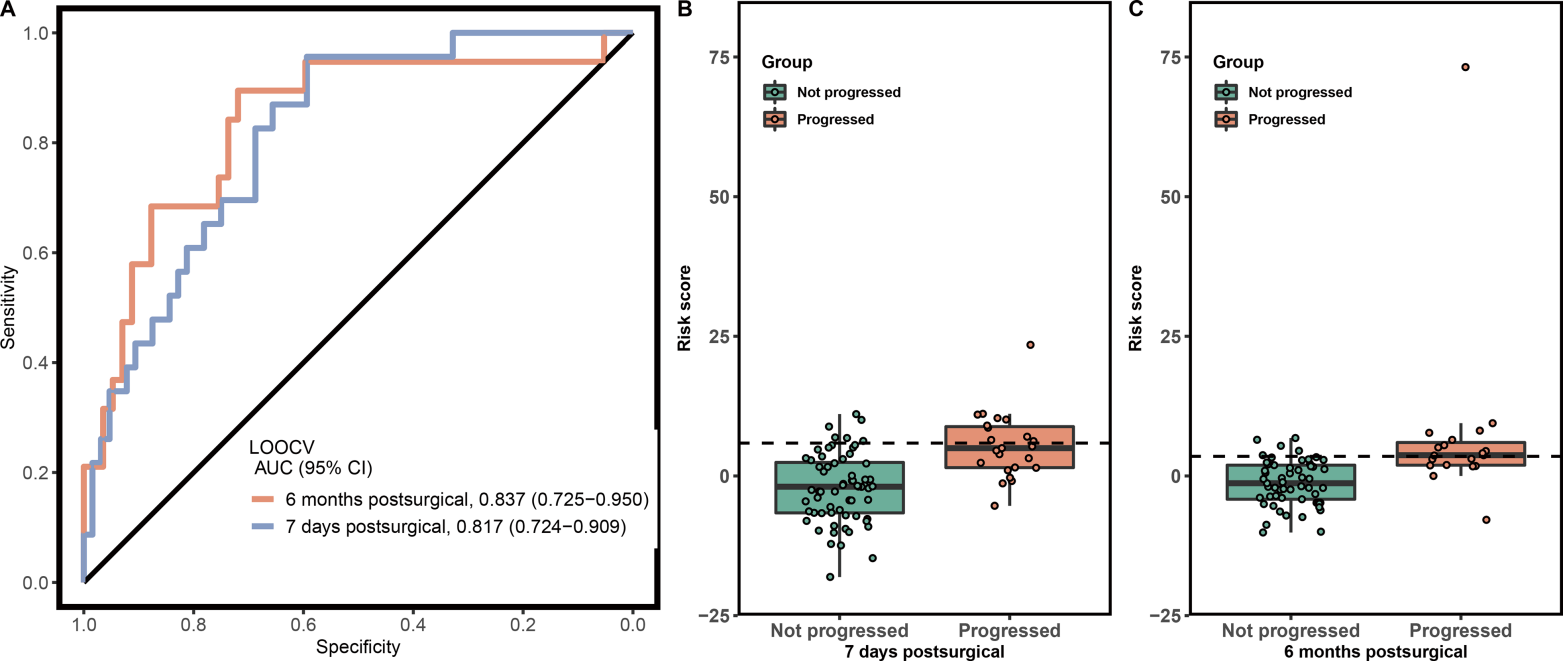
Leave-one-out cross-validation (LOOCV) results of Coxnet model using fragmentomics profiles. **A,** ROC curves for both 7 days and 6 months postsurgical models. **B** and **C,** Barplots for model-predicted risk scores for 7 days and 6 months postsurgical models.

As shown in Fig. 2A, both Coxnet models showed excellent AUCs (7 days postsurgical: 0.817, 95% CI: 0.724–0.909; 6 months postsurgical: 0.837, 95% CI: 0.725–0.950) for distinguishing patients with recurrence from recurrence-free patients. The risk score cutoff for identifying patients with a high risk of recurrence was selected to ensure maximizing sensitivity while maintaining over 90% specificity. As shown in Fig. 2B and C, patients with recurrence showed higher risk scores than recurrence-free patients for both 7 days and 6 months postsurgical models. As shown in Supplementary Table S2, the 7 days postsurgical Coxnet model yielded a sensitivity of 43.5% (95% CI: 23.2–65.5) at 90.6% specificity (95% CI: 80.7–96.5), while the 6 months postsurgical Coxnet model was able to achieve 57.9% sensitivity (95% CI: 33.5–79.7) at 91.2% specificity (95% CI: 80.7–97.1).

**FIGURE 3:**
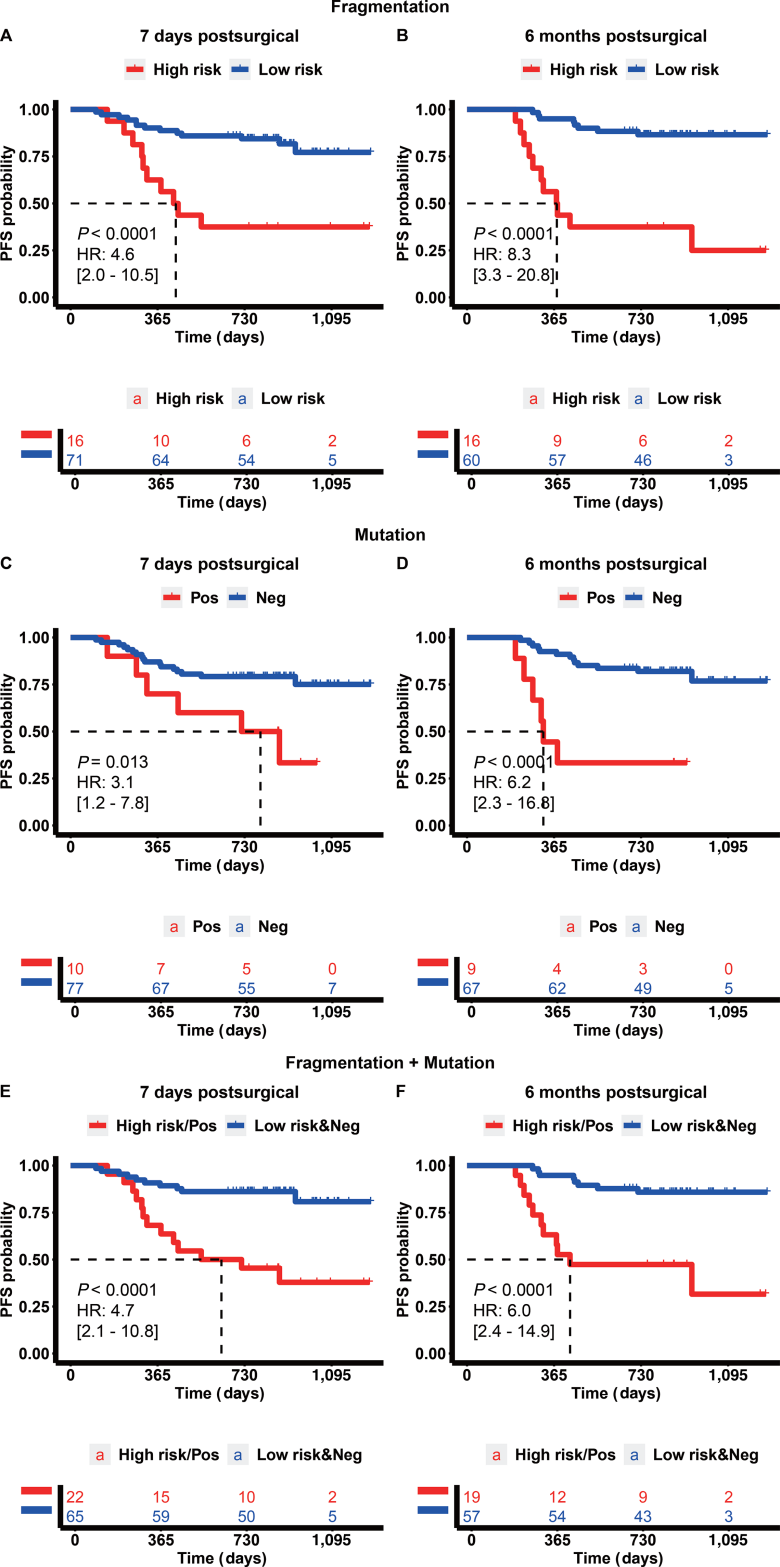
Progression-free survival analysis using fragmentomics model–predicted risk status and ctDNA mutation profile. **A** and **B,** 7 days and 6 months postsurgical prognosis based on fragmentomics model–predicted risk status. **C** and **D,** 7 days and 6 months postsurgical prognosis based on ctDNA mutation profile–predicted risk status. **E** and **F,** 7 days and 6 months postsurgical prognosis based on combination of fragmentomics model and ctDNA mutation profile–predicted risk status. A combined high-risk patient was defined as being model-predicted high risk or ctDNA positive.

At 7 days postsurgical, the high-risk patients predicted by our fragmentomics model showed an increased risk of approximately 4.6 times compared with the low-risk patients (HR = 4.587, 95% CI: 1.998–10.53, *P* < 0.0001; Fig. 3A; Table 1), which is independent from baseline clinical characteristics including age, sex, stage, smoking history, and histology type (multivariate Cox regression, HR = 3.300, 95% CI: 1.228–8.866, *P* = 0.0179). Similarly, the 6 months postsurgical multivariate model showed an increased risk of approximately 8.3 times (HR = 8.285, 95% CI: 3.298–20.81, *P* < 0.0001; Fig. 3B; Table 1). As shown in Table 1, the HR was still significant even after adjusting for baseline clinical characteristics (multivariate Cox regression HR = 6.967, 95% CI: 2.217–21.890, *P* = 0.00089).

**FIGURE 4:**
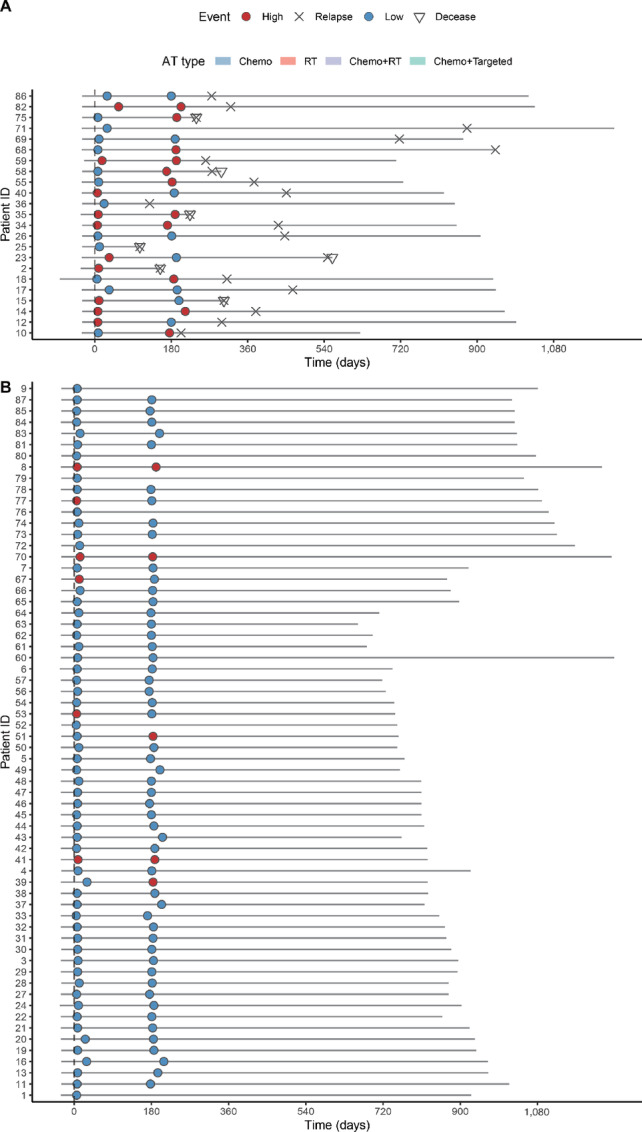
Fragmentomics model prediction ahead of radiographic confirmed recurrence. Swim plot illustrating the fragmentomics model–predicted risk status, adjuvant therapy and pathologic events of cases in which relapse occurred (**A**) and not yet occurred (**B**) during the follow-up.

**FIGURE 5:**
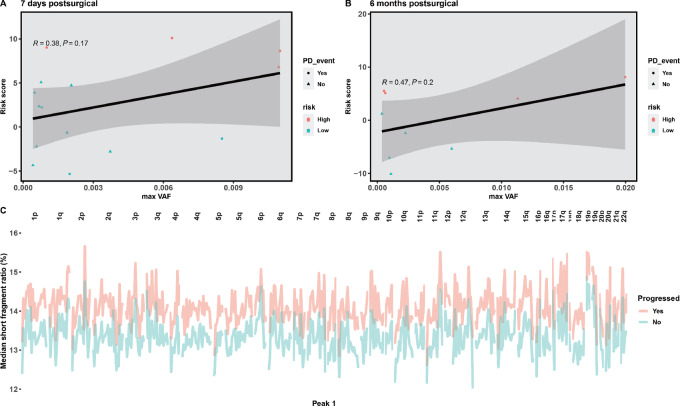
The pattern of cfDNA fragmentomics profiling between progressed and not-yet progressed patients in the cohort. **A** and **B,** Scatter plot showing positive correlation between fragmentomics model–predicted risk scores and ctDNA mutation profile–determined max variant allele frequency (VAF). **C,** Fragmentomics profiles of progressed and not-yet progressed patients with NSCLC at 7 days postsurgical. *Y* axis are the median short to long fragments ratio, *X* axis are the 541 genome-wide bins.

Furthermore, as shown in Fig. 4A and B, 10 patients with recurrence were successfully identified as high risk by the fragmentomics model at 7 days postsurgical. The median lead time was 293 days (145–514 days) for the model-predicted recurrence than the radiographic recurrence. As shown in Fig. 5A and B, the risk scores predicted by the Coxnet models were positively correlated with the max variant allele frequency determined by the ctDNA mutation–based method. Moreover, it is shown that the profiles of short/long fragment ratio at Peak1 were distinguishable between patients who progressed during the follow-up and those who did not yet progress (Fig. 5C). The median short/long ratio was larger in the progressed patient group, indicating that they have shorter cfDNA fragments compared with the yet-to-progress patients. These results were consistent with the previous finding that the plasma cfDNA fragments were aberrantly short in cancer patient samples (25).

### Comparing Fragmentomics Models and ctDNA Mutation Profiling

**FIGURE 6:**
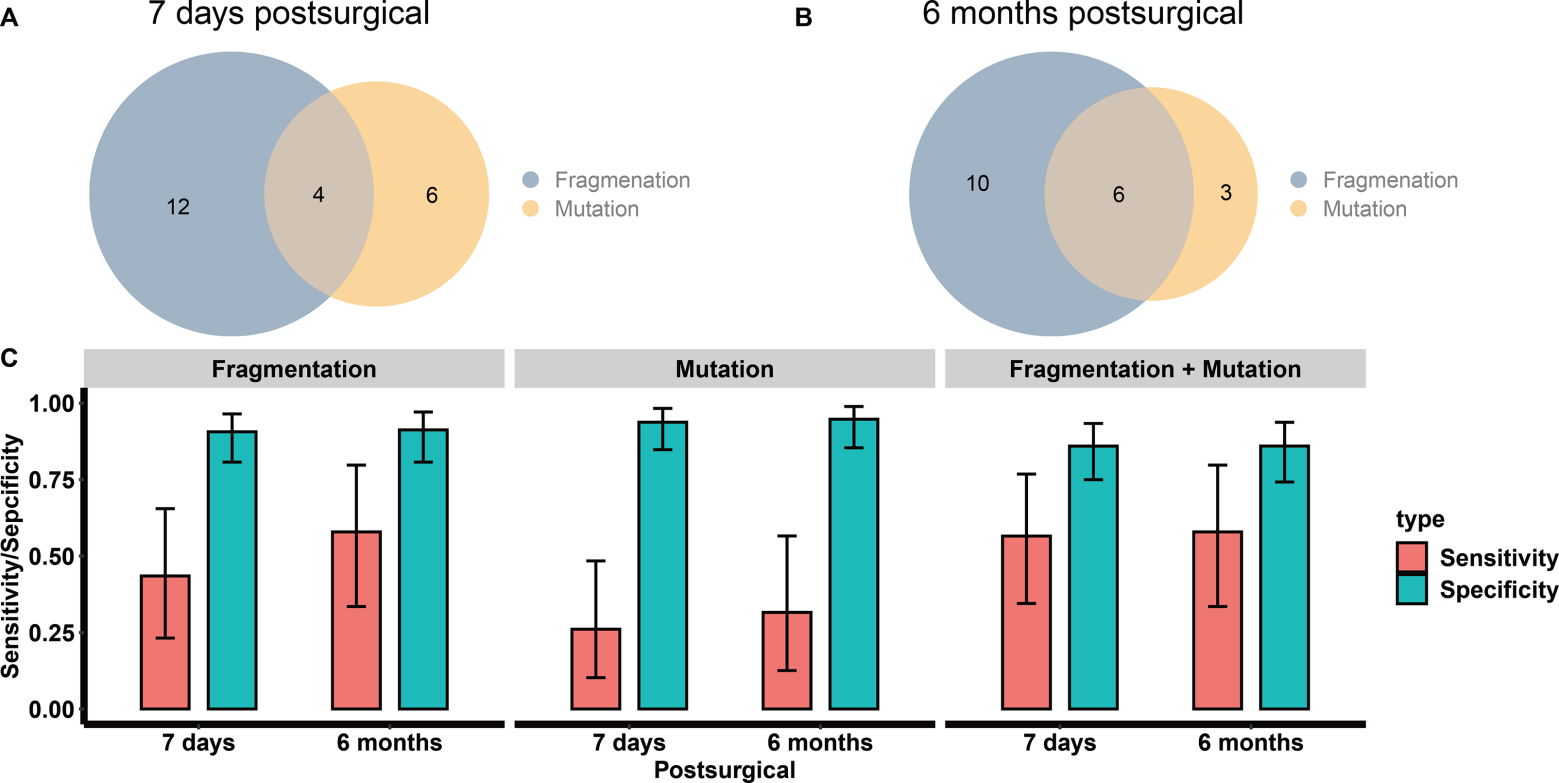
Evaluating fragmentomics model prediction and ctDNA mutation profile status in the cohort. **A** and **B,** Venn diagrams of model prediction and ctDNA status for 7 days and 6 months postsurgical timings. **C,** Barplots of sensitivity and specificity in the cohort. The error bars represent 95% CIs.

We then compared our model-predicted results against the ctDNA mutation profiling results. As shown in Supplementary Table S2, the ctDNA mutation–based method reached a 26.1% sensitivity (95% CI: 10.2–48.4) under 93.8% specificity (95% CI: 84.8–98.3) at 7 days postsurgical and a 31.6% sensitivity (12.6%–56.6%) under 94.7% specificity (95% CI: 85.4–98.9) at 6 months postsurgical. The performances of ctDNA mutation profiling at both 7 days and 6 months postsurgical were inferior to the performances of fragmentomics Coxnet models (Fig. 6). At 7 days postsurgical, the Coxnet model predicted 16 patients with a high risk of recurrence, compared with the only 10 ctDNA-positive patients by mutation profiling, as shown in Fig. 6A. This resulted in a 1.6 times sensitivity for the fragmentomics model at 7 days postsurgical. The same trend was observed at 6 months postsurgical, with the fragmentomics model–predicted 16 patients with high risk of recurrence compared with the 9 ctDNA-positive patients by mutation profiling, yielding a 1.8 times higher sensitivity (Fig. 6B and C).

As shown in Fig. 3C and D, the ctDNA-positive patients showed increased risk of approximately 3.1 (HR = 3.077, 95% CI: 1.212–7.81, *P* = 0.013) and approximately 6.2 times (HR = 6.209, 95% CI: 2.301–20.81, *P* = 0.013) at 7 days and 6 months postsurgical, respectively, which were lower compared with the high-risk patients predicted by the fragmentomics model.

The overall sensitivity was even higher (56.5%, 95% CI: 34.5–76.8) at 7 days postsurgical, when combining the fragmentomics model prediction with the ctDNA mutation profiling results (Fig. 6A and C). A patient was labeled as high risk for progression while having either a high-risk status predicted by the fragmentomics model or a positive ctDNA status based on the target panel sequencing. A low progression risk status would require a patient to have a predicted low-risk status by fragmentomics as well as a negative ctDNA status by mutational profiling (Fig. 3E and F). However, there was no increase for the 6 months postsurgical timepoint, as the sensitivity of combined results was identical for using only fragmentomics model. Furthermore, after combining both 7 days and 6 months postsurgical results, the Coxnet model predicted 24 patients with high risk of recurrence, compared with the 16 ctDNA-positive patients, as shown in Supplementary Fig. S1A. The overall sensitivity reached 78.3% (95% CI: 56.3–92.5) at 79.7% specificity (95% CI: 67.8–88.7) when combining both methods at all timepoints (Supplementary Fig. S1B).

## Discussion

Despite receiving curative surgeries, a large number of patients with NSCLC can still develop recurrence. Therefore, there is an urgent clinical need for MRD detection in patients with postsurgical NSCLC, which can assist in treatment decision-making progress to both maximize the chance of cure and minimize the risk of overtreatment. However, the current MRD detection methods are limited by several significant barriers: low sensitivity and high turnaround time.

Herein, we reported an ultrasensitive assay for enhanced detection of landmark MRD in patients with postsurgical NSCLC. By using the more sensitive WGS approach, which is not limited by the number or specific location of ctDNA molecules, and the newly emerged fragmentomics feature, our Coxnet model was able to predict patients’ MRD status with great sensitivity (Supplementary Figs. S2–S4). At 7 days and 6 months postsurgical, the model-predicted MRD status reached sensitivities of 43.5% (95% CI: 23.2–65.5) and 57.9% (95% CI: 33.5–79.7), at specificities of 90.6% (95% CI: 80.7–96.5) and 91.2% (95% CI: 80.7–97.1), respectively. The fragmentomics models, which outperformed the traditional ctDNA mutation–based method (23%–40% sensitivity), can further enhance the overall sensitivity while combined with ctDNA mutation methods showing its great potential in landmark detection for MRD.

Moreover, as the WGS approach requires no existing knowledge of mutation profile in the cancer tissue samples, the fragmentomics model is, in theory, of more clinical potential by having less turnaround time and limitation due to tumor tissue insufficiency. For example, Li and colleagues 2021 failed to detect any mutation in the baseline plasma sample for approximately 40% of their cohort, contributing to the limited sensitivity of MRD detection (13). One significant advantage of our fragmentomics model is its ability to predict landmark MRD status at a very early stage (7 days) postsurgery compared with other studies (2 weeks to 4 months; refs. 6, 7, 9, 19), which could potentially assist timely decision-making for postsurgery treatment.

However, this study is still limited, especially by the small cohort size and the lack of an independent test cohort. The leave-one-out cross-validation approach, despite having less bias than a K-fold cross-validation approach, can still be subject to overfitting as it relies solely on the training cohort. A large multicenter study is needed to validate the predictive power of cfDNA fragmentomics in landmark MRD detection using an independent validation cohort. We have already started recruiting patients for the independent validation cohort. However, such a cohort is not available in time for this study due to the long follow-up period required to obtain the data. Furthermore, it will be interesting to investigate the effectiveness of postsurgery treatment decision-making assisted by model-predicted MRD status.

## Supplementary Material

Table S1-2 Supplementary Table S1. Patient demography
Supplementary Table S2. Evaluating fragmentomics model performances and ctDNA mutation-based method.

Supplementary Figure S1 Evaluating longitudinal fragmentomics model prediction and ctDNA status

Supplementary Figure S2 Different cross-validation results of Coxnet model using fragmentomics profiles

Supplementary Figure S3 Leave-one-out cross-validation (LOOCV) results of fragmentomics models using different algorithms

Supplementary Figure S4 Recursive feature elimination with cross-validation of Coxnet model using fragmentomics profiles

Supplementary Methods Additional methods for Coxnet models
