# Supplementary material for: Enhanced Detection of Landmark Minimal Residual Disease in Lung Cancer Using Cell-free DNA Fragmentomics
Source: Cancer Res Commun. 2023 May 30;3(5):933–42. doi: 10.1158/2767-9764.CRC-22-0363 (PMC10228550; doi:10.1158/2767-9764.CRC-22-0363)
Supplement: Supplementary Figure S1 — Evaluating longitudinal fragmentomics model prediction and ctDNA status [file crc-22-0363-s02.docx]

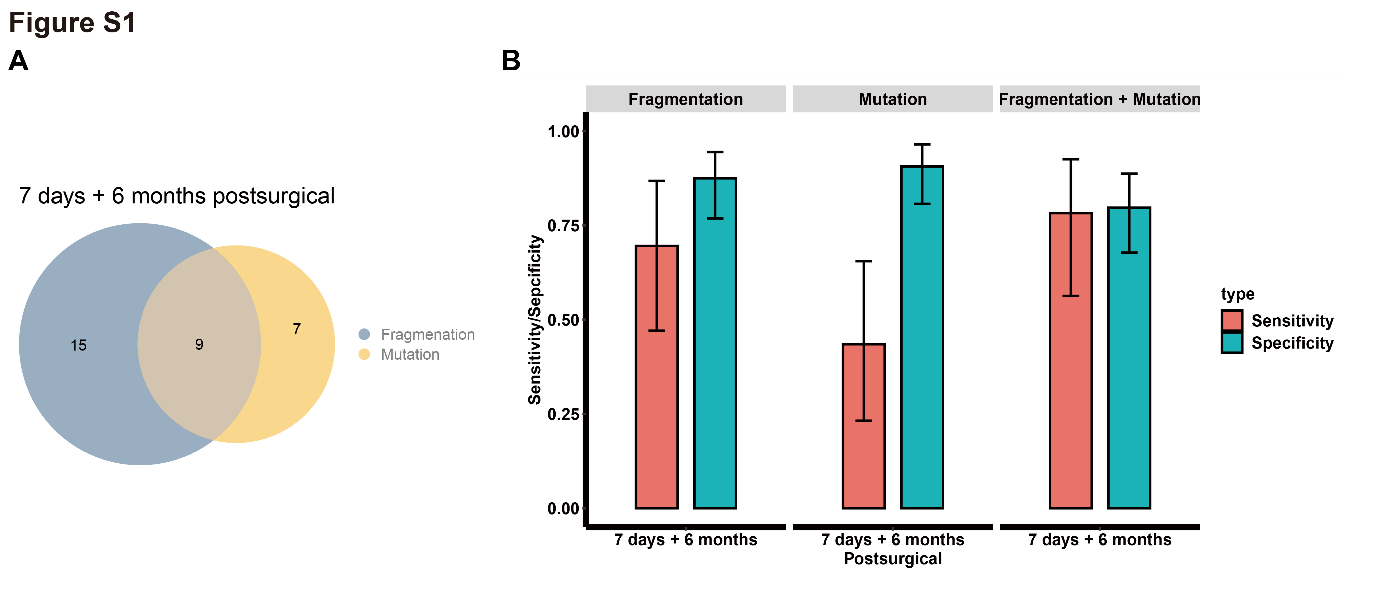


**Supplementary Figure S1**. **Evaluating longitudinal fragmentomics model prediction and ctDNA status.** A) Venn diagrams of model prediction and ctDNA status using combination of 7 days and 6 months postsurgical results. A longitudinal combined high-risk patient was defined as being model predicted high risk or ctDNA positive in either 7 days or 6 months postsurgical. B) Barplots of sensitivity and specificity.
