# Supplementary material for: Enhanced Detection of Landmark Minimal Residual Disease in Lung Cancer Using Cell-free DNA Fragmentomics
Source: Cancer Res Commun. 2023 May 30;3(5):933–42. doi: 10.1158/2767-9764.CRC-22-0363 (PMC10228550; doi:10.1158/2767-9764.CRC-22-0363)
Supplement: Supplementary Figure S2 — Different cross-validation results of Coxnet model using fragmentomics profiles [file crc-22-0363-s03.docx]

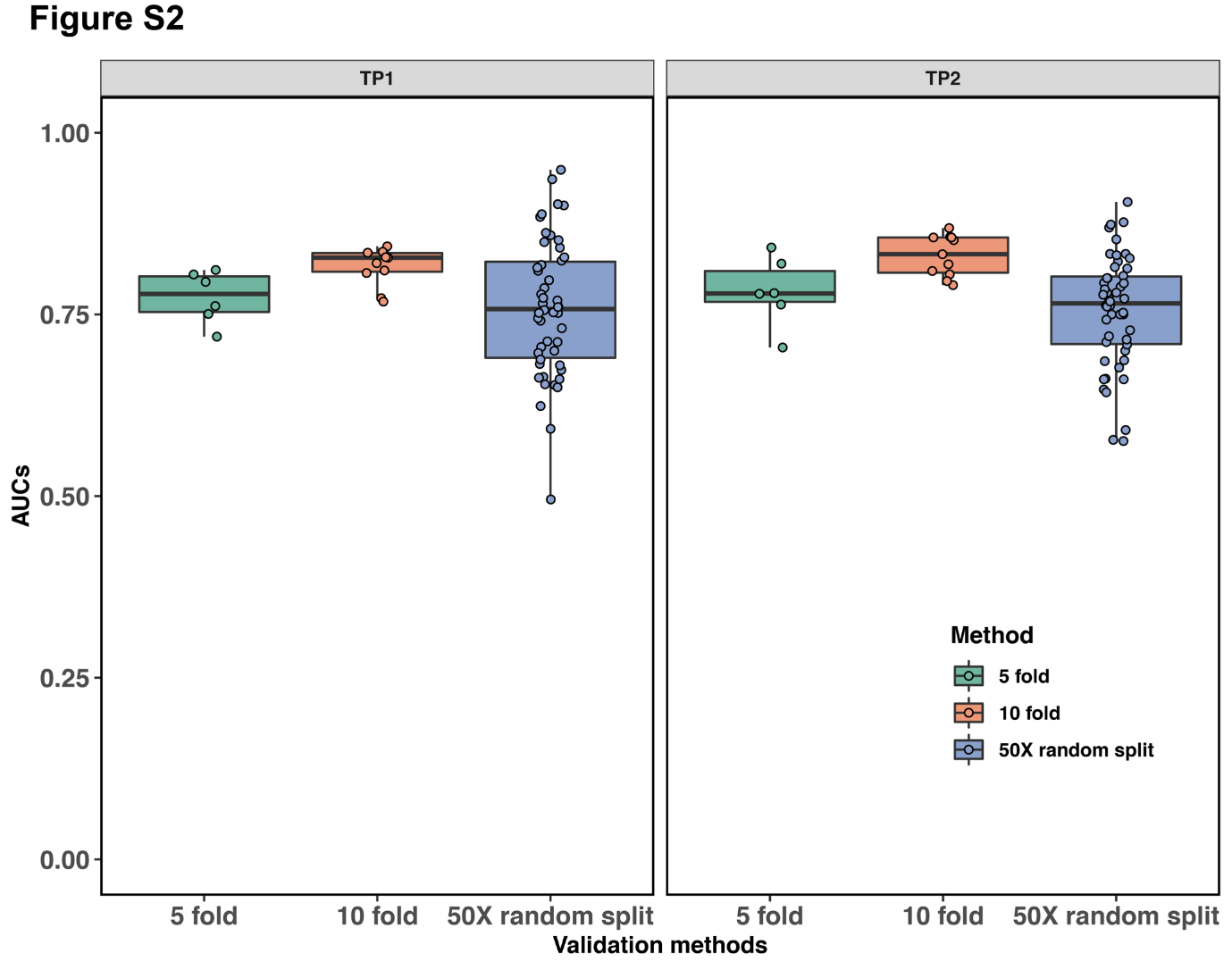


**Supplementary Figure S2. Different cross-validation results of Coxnet model using fragmentomics profiles.** Boxplots of AUCs of 7 days A) and 6 months B) postsurgical models using 5-fold (5 repeats), 10-fold (10 repeats) and 60-40 random splits (50 repeats) cross-validation strategies.
