# Supplementary material for: Enhanced Detection of Landmark Minimal Residual Disease in Lung Cancer Using Cell-free DNA Fragmentomics
Source: Cancer Res Commun. 2023 May 30;3(5):933–42. doi: 10.1158/2767-9764.CRC-22-0363 (PMC10228550; doi:10.1158/2767-9764.CRC-22-0363)
Supplement: Supplementary Figure S3 — Leave-one-out cross-validation (LOOCV) results of fragmentomics models using different algorithms [file crc-22-0363-s04.docx]

**
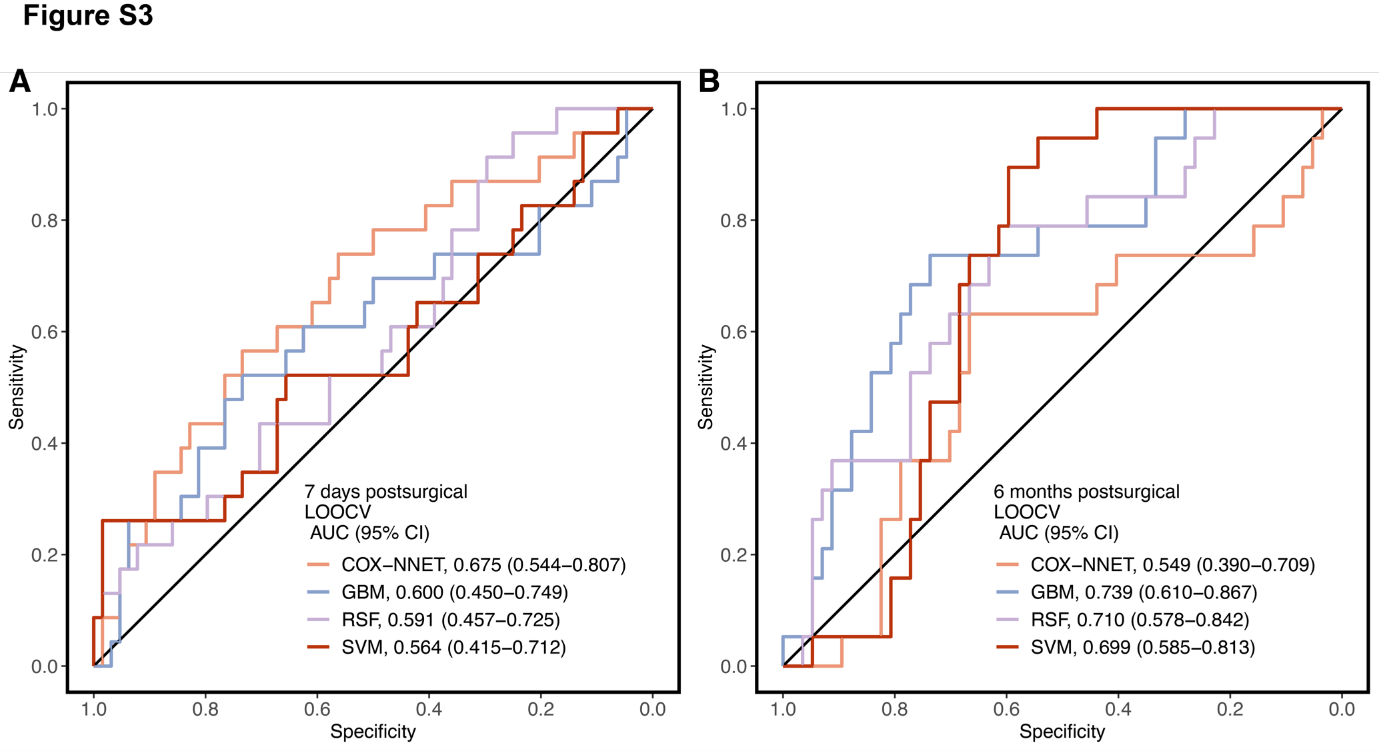
**

**Supplementary Figure S3. Leave-one-out cross-validation (LOOCV) results of fragmentomics models using different algorithms.** A) ROC curves for 7 days postsurgical models constructed by Cox-nnet, GBM, RSF and SVM algorithms. B) ROC curves for 6 months postsurgical models constructed by Cox-nnet, GBM, RSF and SVM algorithms.
