# Supplementary material for: Enhanced Detection of Landmark Minimal Residual Disease in Lung Cancer Using Cell-free DNA Fragmentomics
Source: Cancer Res Commun. 2023 May 30;3(5):933–42. doi: 10.1158/2767-9764.CRC-22-0363 (PMC10228550; doi:10.1158/2767-9764.CRC-22-0363)
Supplement: Supplementary Figure S4 — Recursive feature elimination with cross-validation of Coxnet model using fragmentomics profiles [file crc-22-0363-s05.docx]

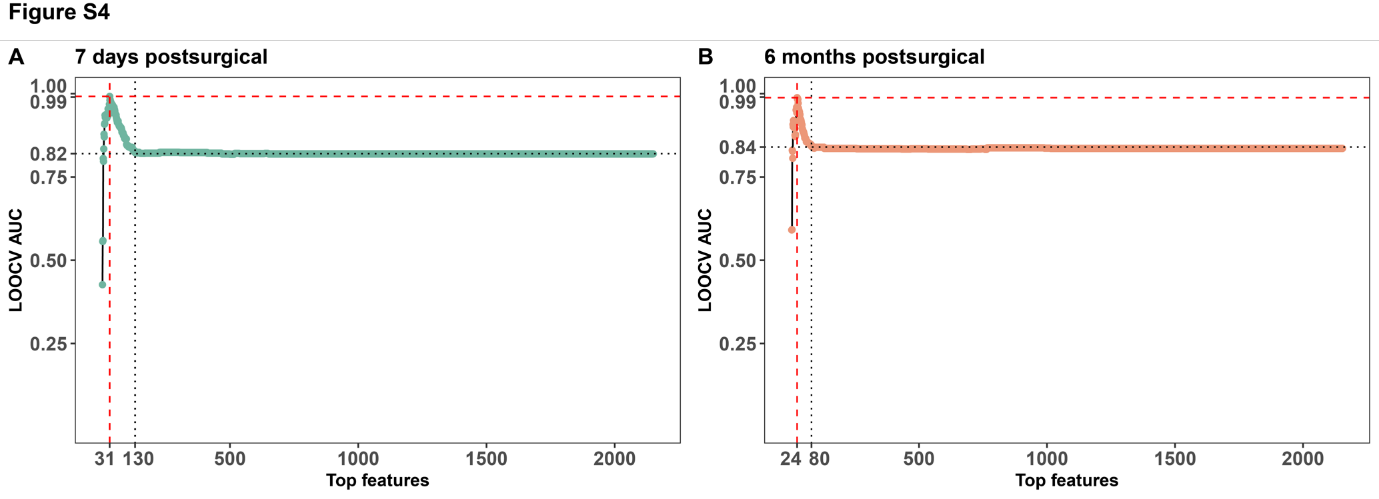


**Supplementary Figure S4.** **Recursive feature elimination with cross-validation of Coxnet model using fragmentomics profiles.** Line plots of 5-fold (5 repeats) RFECV results in the 7 days A) and 6 months B) postsurgical timings.
