# Supplementary material for: Enhanced Detection of Landmark Minimal Residual Disease in Lung Cancer Using Cell-free DNA Fragmentomics
Source: Cancer Res Commun. 2023 May 30;3(5):933–42. doi: 10.1158/2767-9764.CRC-22-0363 (PMC10228550; doi:10.1158/2767-9764.CRC-22-0363)
Supplement: Supplementary Methods — Additional methods for Coxnet models [file crc-22-0363-s06.docx]

**Title:** Enhanced Detection of Landmark Minimal Residual Disease in Lung Cancer using Cell-Free DNA Fragmentomics

**Authors:** Wang et al.

**Supplementary methods**

**Multiple cross-validations of Coxnet models**

A total of 87 NSCLC patients, including 23 patients with recurrence during follow-up, were used to construct predictive models for MRD. Two machine learning models, which used either 7 days or 6 months of postsurgical samples, were constructed based on the Penalized Cox Models (Coxnet) algorithm by the scikit-survival package (0.17.2) (1). Both models used three more cross-validation methods (5 times repeated 5-fold cross-validation, 10 times repeated 10-fold cross-validation, 50 times random split [60% to 40%]) to evaluate the predictive performance of MRD, in addition to the leave-one-out cross-validation.

The cohort samples were randomly shuffled and split into K-folds for the repeated K-fold cross-validation (5 times repeated 5-fold cross-validation, 10 times repeated 10-fold cross-validation). For each unique K-fold as the test set, a model was fitted using the remaining K-1 folds as the training set, which was used for evaluating the test set. Receiver operating characteristic (ROC) curves were constructed by the pROC package (v. 1.17.0.1) using the sample risk scores for each K-fold cross-validation and the average risk scores for the N times repeats.

For each of the 50 times random split cross-validation, the cohorts were split into a training set (60%) and a test set (40%) after being randomly shuffled. A model was fitted using the training set, and risk scores were obtained using the test set. ROC curves were constructed for each of the 50 times random split cross-validation.

The different cross-validating methods showed satisfactory performance levels compared to the leave-one-out cross-validation, as shown in **Figure S2**. The 5 times repeated 5-fold cross-validation showed excellent AUCs in both the 7 days postsurgical cohort (0.811 [average risk scores], 0.719-0.805) and the 6 months postsurgical cohort (0.820 [average risk scores], 0.705-0.842). The 10 times repeated 10-fold cross-validation showed even higher AUCs in both the 7 days postsurgical cohort (0.835 [average risk scores], 0.768-0.844) and the 6 months postsurgical cohort (0.856 [average risk scores], 0.790-0.869). The 50 times random split cross-validation reached an average AUC of 0.758 (0.495-0.949) and 0.755 (0.576-0.905) in the 7 days postsurgical cohort and the 6 months postsurgical cohort, respectively.

**Comparing Coxnet against other algorithms**

We also evaluated the Coxnet algorithm against other algorithms, including Gradient Boosted Models (GBM), Random Survival Forests (RSF) and Survival Support Vector Machine (SVM) by the same scikit-survival package (0.17.2) (1), as well as Cox-nnet (2).

A total of 87 NSCLC patients, including 23 patients with recurrence during follow-up, were used to construct predictive models for MRD. Machine learning models were constructed using the 4 algorithms (Cox-nnet, GBM, RSF, SVM) on the 7 days or 6 months of postsurgical samples. The same leave-one-out cross-validation strategy was implemented to evaluate the predictive performance of MRD. Similarly, each sample (N = 87 and 76) was used once as a validation set during the leave-one-out cross-validation. The remaining samples (86 or 75) were used as the training set to fit the model, which was then used for predicting the MRD risk score for the validation set. This process was then repeated 87 (76) times until every sample’s risk score was generated, which was then used to generate ROC curves for all algorithms.

As shown in **Figure S3A**, the 4 models (Cox-nnet, GBM, RSF and SVM) all demonstrated poorer AUCs (0.564-0.675) compared to the Coxnet model (0.817) in the 7 days postsurgical cohort. A similar pattern was also observed in the 6 months postsurgical cohort, as the Cox-nnet (AUC: 0.549), GBM (AUC: 0.739), RSF (AUC: 0.710), and SVM (AUC: 0.699) models showed inferior performance compared to the Coxnet (AUC: 0.837) model (**Figure S3B**).

**Feature importance for the Coxnet models**

We performed recursive feature elimination leave-one-out cross-validation to investigate the contribution of different cfDNA fragmentomics features on MRD detection. The cfDNA fragmentomic feature coefficients were retrieved from all the Coxnet models (N = 87 and 76) during the leave-one-out cross-validation (LOOCV) and ranked by the absolute value from highest to lowest. The importance for these 2,164 features were determined by their average rank. A recursive feature elimination LOOCV was performed by eliminating the least important feature, and the AUCs using different feature combinations were retrieved and plotted.

As illustrated in **Figure S4**, the AUCs of the recursive feature elimination cross-validation plateaued (0.82 and 0.84) after using top 130 and 80 features in the 7 days and 6 months postsurgical cohort, respectively. Both cohorts reached a maximum AUC of 0.99 while using the top 31 and 24 features, however, such high AUCs could be the result of small cohort size and the absence of a true independent test cohort.

**Supplementary references**

1. Pölsterl S. scikit-survival: A Library for Time-to-Event Analysis Built on Top of scikit-learn. Journal of Machine Learning Research **2020**;21:1-6

2. Wang D, Jing Z, He K, Garmire LX. Cox-nnet v2.0: improved neural-network based survival prediction extended to large-scale EMR data. Bioinformatics **2021**;37:2772-4
